# Combining angioplasty with percutaneous microwave ablation for treating primary Budd-Chiari syndrome associated with hepatocellular carcinoma in two patients: A case report

**DOI:** 10.3892/ol.2013.1417

**Published:** 2013-06-20

**Authors:** QING-QIAO ZHANG, MAO-HENG ZU, HAO XU, YU-MING GU, WEN-LIANG WANG, ZHI-KANG GAO

**Affiliations:** Department of Interventional Radiology, The Affiliated Hospital of Xuzhou Medical College, Xuzhou, Jiangsu 221006, P.R. China

**Keywords:** Budd-Chiari syndrome, hepatocellular carcinoma, angioplasty, percutaneous microwave ablation

## Abstract

Percutaneous transluminal angioplasty using balloon catheters for Budd-Chiari syndrome (BCS) and transcatheter arterial chemoembolization (TACE) for unresectable hepatocellular carcinoma (HCC) have become increasingly accepted as alternative therapeutic modalities. However, few studies have investigated the clinical efficacy of combining percutaneous microwave ablation with angioplasty for patients with BCS complicated by HCC. In the present study, a safe and effective method for treating BCS associated with HCC is presented. Color Doppler ultrasonography, magnetic resonance imaging (MRI), computed tomography (CT), inferior venacavography, hepatic arteriogram and cytological examinations were used for the diagnosis. A KY2000 microwave system with an emission of 915 MHz was also employed for the treatment. Two patients with BCS associated with HCC that were administered different adjuvant drug treatments underwent percutaneous transluminal angioplasty and percutaneous microwave ablation successfully, with no treatment-related complications. Combining angioplasty with percutaneous microwave ablation may represent an alternative method for the treatment of BCS associated with HCC.

## Introduction

Primary Budd-Chiari syndrome (BCS) is a rare clinical entity characterized by a blocked hepatic venous outflow tract at various levels from the small hepatic veins to the inferior vena cava (IVC) (1,2). Hepatocellular carcinoma (HCC) is one of the major complications of BCS (3,4). The incidence of HCC associated with BCS varies between different case studies. In Korea, France and Japan, 23/159 (14.5%), 11/97 (11.3%) and 3/12 (25%) BCS patients, respectively, were reported to exhibit complications as a result of HCC (4–6).

Resection surgery, systemic chemotherapy, target therapy with sorafenib, radiotherapy and transcatheter arterial chemoembolization have been reported as therapeutic modalities for HCC patients with main portal vein invasion or BCS (3,7,8). However, the long-term outcome is poor, with a median survival of 2–20 months despite therapeutic treatment (3,7,8). Percutaneous transluminal angioplasty using balloon catheters for BCS and transcatheter arterial chemoembolization (TACE) for unresectable HCC are being increasingly reported as suitable alternative therapeutic modalities (9,10). Few studies have investigated the clinical efficacy of combining percutaneous microwave ablation with angioplasty for patients with BCS complicated by HCC. The current study presents two cases of BCS associated with HCC within a 4-month period that were treated with percutaneous microwave ablation and angioplasty. Written informed consent was obtained from the patients.

## Case report

### Patient 1

**Figure 1.**
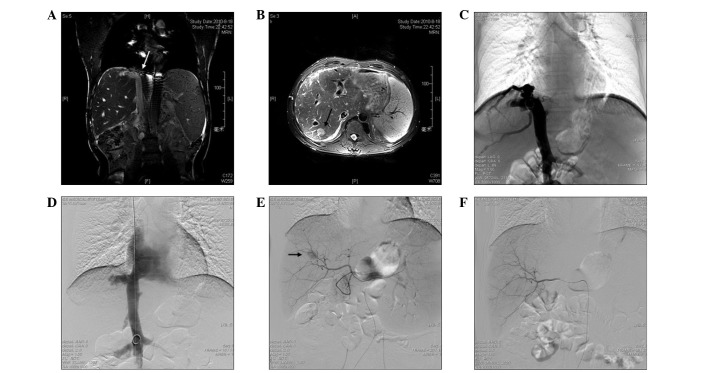
Diagnosis and treatment images of patient 1. (A) Coronal source image obtained from magnetic resonance angiography demonstrates membranous obstruction of the IVC (arrow). (B) T2-weighted MRI reveals a homogeneously hypertensive area (arrow). (C) Inferior vena cavagram illustrates membranous obstruction. (D) Inferior vena cavagram performed immediately following balloon dilation indicates full IVC patency. (E) Common hepatic angiogram reveals a hypervascular mass (arrow) supplied by segment VII of the hepatic artery. (F) Right hepatic angiogram from selective TACE performed through the feeding artery reveals successful embolization of vessels supplying the tumor. IVC, inferior vena cava; MRI, magnetic resonance imaging; TACE, transcatheter arterial chemoembolization.

A 43-year-old male with a history of abdominal wall veins varices for 15 years and hepatosplenomegaly and abdominal distension for 3 months was admitted to the Affiliated Hospital of Xuzhou Medical College (Xuzhou, China). The patient reported no past or present alcohol consumption or a history of diabetes or hepatitis. At the time of presentation, the patient’s routine laboratory test results were as follows: Hemoglobin (Hb), 144 g/l (normal range, 120–160 g/l); white blood cells, 1.7×10^9^ cells/l (normal range, 4–10×10^9^ cells/l); platelet count, 27×10^9^ cells/l (normal range, 100–300×10^9^ cells/l); total protein, 78 g/l (normal range, 60–80 g/l); serum albumin, 40.3 g/l (normal range, 34–55 g/l); serum bilirubin, 30.3 *μ*mol/l (normal range, 0–20 *μ*mol/l); aspartate aminotransferase (AST), 26 U/l (normal range, 0–40 U/l); alanine aminotransferase (ALT), 25 U/l (normal range, 0–40 U/l); γ-glutamyl transpeptadase (GGT), 22 U/l (normal range, 0–40 U/l); and alkaline phosphatase (ALP), 71 U/l (normal range, 42–128 U/l). The α-fetoprotein (AFP) level was 428 ng/ml (normal range, 0–20 ng/ml). The results also included negative hepatitis B and C viral serologies. Imaging studies included color Doppler ultrasonography, magnetic resonance imaging (MRI), computed tomography (CT), inferior venacavography and hepatic arteriograms. These studies indicated the membranous obstruction of the IVC (BCS) (Fig. 1A), an enlarged caudate lobe, patent hepatic and portal veins, azygos and hemiazygos vein varices and a 2.2×1.7×1.4-cm hypervascular mass, with washout during the portal venous phase in the superior segment of the right hepatic lobe, consistent with segment VII (Fig. 1B). The patient was diagnosed with membranous obstruction of the IVC, associated with HCC due to elevated levels of AFP and the typical findings on MRI and CT.

Angioplasty was the first procedure to be performed and informed written consent was obtained prior to this. A 5F sheath was advanced through a percutaneous right femoral vein, then a 5F pigtail catheter (Cook, Inc., Bloomington, IN, USA) was inserted over a 0.035-inch guide wire into the IVC, followed by the inferior vena cavography to confirm the obstruction (Fig. 1C). The 5F pigtail catheter was inserted trans-femorally into the distal area of the obstruction as the marker for positioning and a J-type Brockenbrough needle (Cook, Inc.) was introduced into the proximal region of the obstruction, using the right jugular vein to cut through the lesion under the fluoroscopic guidance in the optimal view. When the obstruction was broken, the needle was exchanged for a 4F catheter. A 260-cm ultra-stiff guide wire was inserted through the 4F catheter and the catheter was withdrawn. Following this, a balloon catheter (28–50 mm; Cordis Corporation, Miami, FL, USA) was inserted to dilate the IVC obstruction twice. Inferior vena cavography was performed immediately following dilation and revealed good flow into the atrium (Fig. 1D).

**Figure 2.**
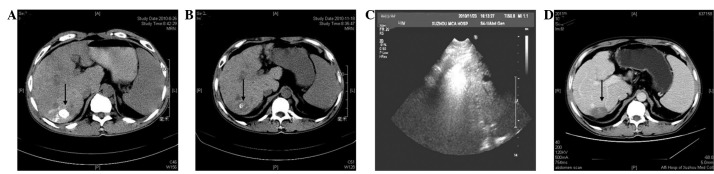
Diagnosis images of patient 1 at follow-up. (A) Axial non-contrast CT image obtained one week after TACE reveals complete iodized oil retention inside the HCC (arrow). (B) Axial non-contrast CT image obtained 3 months after TACE reveals the almost complete disappearance of iodized oil inside the HCC (arrow). (C) Sonogram obtained during sonographically-guided percutaneous microwave ablation. (D) Axial contrast-enhanced CT scan obtained 9 months after microwave ablation reveals an area of hypoattenuation without enhancement, indicative of a complete response (arrow). TACE, transcatheter arterial chemoembolization; CT, computed tomography; HCC, hepatocellular carcinoma.

Although the tumor was resectable, the patient refused surgical resection and was allowed to proceed with TACE. A celiac arteriography was initially performed to assess the anatomy, tumor burden and vascularity (Fig. 1E). Selective catheterization of the segmental branch of the right hepatic artery, which was feeding the lesion, was then performed using a 2.9F microcatheter (SP). A mixture of 5 ml iodized oil (Lipiodol; Laboratoire Guerbet, Aulnay-Sous-Bois, France) and 10 mg pirarubicin hydrochloride was infused into the feeding artery, followed by selective arterial embolization using gelatin sponge particles. Angiography was performed immediately and revealed that the feeding artery had caused the development of an emboli (Fig. 1F). One week later at follow-up, a non-contrast CT scan revealed that the complete iodized oil had been retained inside the tumor (Fig. 2A) and that the serum levels of AFP had decreased to 26.3 ng/ml. Three months later at follow-up, a non-contrast hepatic CT scan indicated that the iodized oil deposit was almost washed out (Fig. 2B) and that the serum levels of AFP had increased to 112 ng/ml. Further treatment was required and percutaneous microwave ablation was scheduled. A KY2000 microwave system with an emission of 915 MHz (Kangyou Medical Microwave Institute, Nanjing, China) was used on the patient. The system was equipped with 15-gauge needle electrodes (diameter, 1.8 mm and length, 20 cm), which were specifically coated and insulated to prevent tissue adhesion and had internally cycling water to cool the pole to avoid burning the skin. Prior to treatment, an appropriate puncture route was selected for ultrasound. A single antenna was then inserted percutaneously into the tumor and located at the designated sites under ultrasound guidance. A power output setting of 60 W for 300 sec was used during the ablations (Fig. 2C). Nine months later, contrast-enhanced CT imaging results revealed no areas of contrast material enhancement in the lesion following microwave ablation (Fig. 2D). During 24 months of follow-up, the patient was free of symptoms; the IVC was patent and the AFP serum levels and liver function test results were normal.

### Patient 2

**Figure 3.**
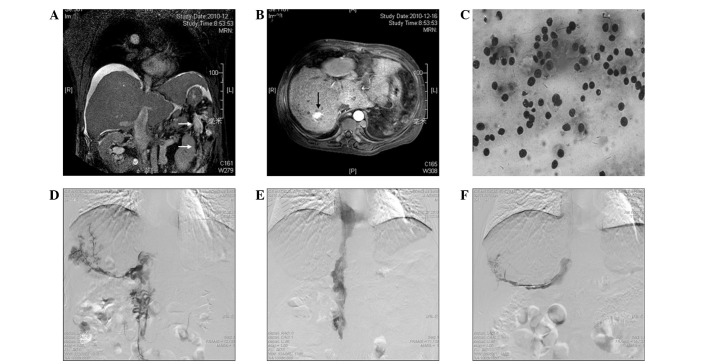
Diagnosis and treatment images of patient 2. (A) Coronal source image obtained from magnetic resonance angiography demonstrates segmental obstruction of the IVC (arrows) and mild ascites. (B) Axial arterial phase contrast-enhanced MRI reveals hypervascular HCC at segmental VII (arrow). (C) Representative image of histopathological observation for liver cancer cells (HE staining; magnification, ×400). (D) Digital subtraction angiography reveals obstruction of the IVC and three hepatic veins, massive thrombosis in the IVC and right accessory hepatic vein. (E) Following balloon dilation, the IVC was clearly visible with chronic thrombosis and stenosis at the intrahepatic portion. (F) On day 19, the right accessory hepatic vein was almost clearly visible by angiography imaging. IVC, inferior vena cava; MRI, magnetic resonance imaging; HCC, hepatocellular carcinoma.

A 56-year-old male patient with a 12-year history of vein varices on the abdominal wall and lower extremities, leg pigmentation for 10 years and leg ulcers for 2 years was admitted to the Affiliated Hospital of Xuzhou Medical College. The individual was treated by splenectomy in another hospital due to hypersplenism 1 month prior to admission, but experienced no clinical improvement. Seven days prior to admission to the Affiliated Hospital of Xuzhou Medical College, the patient began to complain of pain and swelling in the right lower extremity. Upon admission, the routine laboratory tests results were as follows: Hb, 125 g/l; white blood cells, 4.97×10^9^ cells/l; platelet count, 259×10^9^ cells/l; total protein, 70.6 g/l; serum albumin, 30.4 g/l; serum bilirubin, 20.5 *μ*mol/l; AST, 53 U/l; ALT, 15 U/l; GGT, 95 U/l; and ALP, 176 U/l. The serum AFP levels were 6.7 ng/ml. Color Doppler ultrasonography and MRI revealed ascites and BCS (segment obstruction of the IVC and three hepatic veins, as well as massive thromboses in the IVC), associated with HCC due to elevated levels of AFP (Fig. 3A). Magnetic resonance venography revealed a 2.3×2.0×1.5-cm well-demarcated and hypervascular tumor located in segment VII of the liver (Fig. 3B). A biopsy confirmed the diagnosis of HCC (Fig. 3C).

A thrombolysis catheter was placed into the right accessory hepatic vein and IVC through the left femoral vein (Fig. 3D). A bolus of 10 ml mixed urokinase (10,000 units) was injected every 4 h to dissolve the thrombus with full-dose heparin. Serial venography revealed gradual resolution of the clot in the right accessory hepatic vein and IVC, however, a large amount of thrombus remained in the iliac and femoral veins. On day 6, a 5F pigtail catheter was inserted into the distal area of the IVC obstruction through the left femoral vein as a marker for positioning; a J-type Brockenbrough needle (Cook, Inc.) was introduced into the proximal section of the IVC obstruction via the right jugular vein to cut through the lesion under fluoroscopic guidance. Following the rupture of the IVC occlusion, a 5F thrombolysis catheter was inserted into the right femoral vein through the IVC. Thrombolysis was continued over the course of the next 4 days. Following complete resolution of the fresh thrombus in the right accessory hepatic vein, IVC and right iliac and femoral veins, a 25–50-mm balloon catheter (Cook, Inc.) was inserted and located at the segmental obstruction of the IVC. The balloon was then dilated to obtain full expansion of the IVC. Angiography of the IVC and iliofemoral vein was performed immediately following the procedure. A patent IVC (Fig. 3E) and right iliofemoral vein was observed following angioplasty. Due to an area of chronic thrombosis and stenosis, which was observed at the intrahepatic portion of the IVC and the right accessory hepatic vein, catheter-directed thrombolysis was continued over the next 9 days. On day 19, the right accessory hepatic vein was almost clearly visible upon angiography (Fig. 3F). The patient received a total dose of 11.4×10^6^ units urokinase over the 19 days.

**Figure 4.**
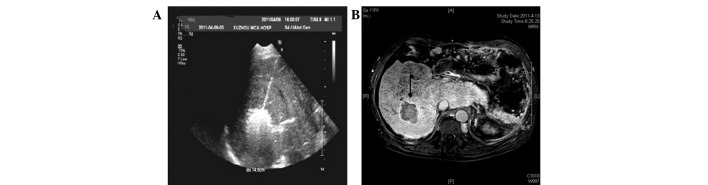
Diagnosis image of patient 2 at follow-up. (A) Sonogram obtained during sonographically-guided percutaneous microwave ablation. (B) Contrast-enhanced MRI image obtained following percutaneous microwave coagulation therapy reveals a hypointense area with hyperintense rim (arrow). MRI, magnetic resonance imaging.

As the patient refused to undergo resection or orthotopic liver transplantation, percutaneous microwave ablation was performed. The treatment was performed under ultrasound guidance with the patient under intravenous anesthesia. The microwave unit used in this case was the same type as in patient 1. An appropriate puncture route was selected for ultrasound, and local anesthesia with 1% lidocaine was administered. Next, a single antenna was percutaneously inserted into the tumor and placed at designated sites under ultrasound guidance. A power output of 40 W for 120 sec, 50 W for 420 sec and 60 W for 60 sec was used during microwave ablation (Fig. 4A). No major complications occurred during or following the surgery. Gadolinium-enhanced MRI performed one week after the percutaneous microwave coagulation therapy revealed a hypointensive area with a hyperintensive rim and unenhanced area within the treated region (Fig. 4B). During the 16 months of follow-up, imaging revealed no recurrence and the patient’s liver function results were almost normal.

## Discussion

The current study presents two cases of primary BCS complicated by HCC that were successfully treated with percutaneous transluminal angioplasty and percutaneous microwave ablation, without any complications. TACE was used to treat the HCC in one case and catheter-directed thrombolysis was used to treat accessory hepatic vein, IVC and lower extremity thromboses in the other case.

There are three main types of BCS: Type I, occlusion of the IVC; type II, occlusion of the hepatic veins; and type III, occlusion of the IVC and the hepatic veins. The incidence of HCC combined with BCS varies between the types of BCS. Type I BCS is more prone to inducing HCC and the incidence ranges between 10.7 and 43.5% (6,11–14). In the present case study, the two patients suffered from BCS combined with HCC. Patient 1 was of type I and patient 2 was of type III BCS. To date, the underlying mechanisms involved in HCC induction by BCS remain to be determined. In a previous study, Shrestha hypothesized that hepatic vena cava disease is an independent risk factor of HCC (14).

BCS combined with HCC is different from benign regenerative nodules of the liver in BCS. Brancatelli *et al* (15) previously demonstrated that the diameters of benign regenerative nodules in the liver are smaller (0.5–4 cm) than HCCs; the quantity of nodules were higher and they were distributed diffusely in the left and right lobe of liver, the major lesion density was higher than the ambient normal hepatic tissues, as indicated by CT plain scans. Large regenerative nodules were bright on T1-weighted magnetic resonance images and presented the same enhancement characteristics following intravenous bolus administration of gadolinium contrast material. Vilgrain *et al* (16) analyzed 23 cases of liver nodules in BCS, as confirmed by pathohistology. Specifically, 4 cases had an average maximal diameter of 7.3 cm for HCC lesions and a quantity of 1–3 lesions; 19 cases exhibited benign regenerative nodules with an average diameter of maximal lesions of 3.3 cm. The quantity of nodules was high, with >10 in 15 cases. In the present case study, although the diameters of the liver nodules were <3 cm in the two patients, all nodules were solitary. The final diagnosis of patient 1 was based on the results of CT, digital subtraction angiography (DSA) and AFP (>400 ng/mL). Patient 2 was diagnosed with HCC based on the results of the cytological examination.

Angioplasty is widely accepted as the main treatment procedure for BCS (9,17). Satisfactory results may be achieved using thrombolytic therapy for the occlusion of the IVC or the hepatic veins (18). Balloon dilation in the IVC was conducted in one case and thrombolysis by catheter and balloon dilation in the IVC was conducted in the other. In the two cases, the IVC was unblocked successfully and complications did not occur.

The therapeutic treatment of BCS combined with HCC includes TACE and surgery. Gwon *et al* (5) reported survival rates of 3 and 5 years in 64 and 50.4% of cases, respectively, when using TACE for the treatment of BCS combined with HCC. Following the administration of TACE to patient 1, reduced iodized oil was found in the HCC lesions during the follow-up. Therefore, percutaneous microwave ablation was considered to be a suitable treatment. A radical cure may be achieved in smaller HCCs (≤4 cm) by employing percutaneous microwave ablation. The 5-year survival rate for this technique has been shown to be similar to that in patients undergoing surgical treatment (19, 20). In patient 1 and 2, the average diameter of the HCC nodules was <3 cm, which was suitable for percutaneous microwave ablation. No recurrence was identified in patients 1 and 2 during 24 and 16 months of follow-up, respectively, indicating the efficacy of this treatment.

In the two present cases, angioplasty was performed followed by percutaneous microwave ablation; following removal of the IVC or hepatic vein blockage, liver congestion was relieved and the absorption of the ascites was facilitated. This protocol prevents complications, including liver hemorrhage, which may be caused by percutaneous microwave ablation.

Objectively, the limitations of this protocol were as follows: i) The number of cases in the present study is low; and ii) the long-term effects of the protocol remain to be observed. However, in general, the combination of angioplasty with percutaneous microwave ablation is likely to represent a safe and effective method for treating BCS associated with HCC.
